# Construction of Ni_2_P-MoC/Coal-Based Carbon Fiber Self-Supporting Catalysts for Enhanced Hydrogen Evolution

**DOI:** 10.3390/molecules29010116

**Published:** 2023-12-24

**Authors:** Xinyue Jia, Mengran Lou, Yang Wang, Ruiying Wang

**Affiliations:** State Key Laboratory of Chemistry and Utilization of Carbon Based Energy Resources, College of Chemistry, Xinjiang University, Urumqi 830017, China; xinyuejia@stu.xju.edu.cn (X.J.); 107556520090@stu.xju.edu.cn (M.L.); 107556523137@stu.xju.edu.cn (Y.W.)

**Keywords:** molybdenum carbide, nickel phosphide, coal-based carbon fibers, hydrogen evolution reaction, electrocatalysis

## Abstract

Efficient and inexpensive electrocatalysts play an important role in the hydrogen evolution reaction (HER) of electrolytic water splitting. Herein, Ni_2_P-MoC/coal-based carbon fiber (Ni_2_P-MoC/C-CF) self-supporting catalysts were obtained by low-temperature phosphorization and high-temperature carbonization. The Mo source and oxidized coal were uniformly dispersed in the carbon support by electrospinning technology. A precursor of Ni was introduced by the impregnation method. The synergistic effect of MoC and Ni_2_P may reduce the strong hydrogen adsorption capacity of pure MoC and provide a fast hydrogen release process. In addition, the C-CFs prepared by electrospinning can not only prevent the agglomeration of MoC and Ni_2_P particles at a high temperature but also provide a self-supporting support for the catalyst. As a result, the catalytic performance of the HER was improved greatly, and a low overpotential of 112 mV at 10 mA cm^−2^ was exhibited stably by the Ni_2_P-MoC/C-CFs. This work not only converts coal into coal-based carbon materials but also provides a feasible pathway for the rational design of large-scale molded hydrogen electrocatalysts.

## 1. Introduction

The global use of fossil fuels is increasing year by year, and a large amount of greenhouse gas emissions has led to a series of increasingly significant problems such as environmental pollution, energy depletion, and global warming [1]. There is an urgent need to develop sustainable clean energy to meet development needs [2]. Hydrogen energy, an important alternative energy for carbon-based fuel that is clean, efficient, and environment-friendly, plays a key role in green energy and chemical conversions [3]. The reaction involved in the electrolysis of water consists of two half-reactions: the hydrogen evolution reaction (HER) and the oxygen evolution reaction (OER) [4]. Among them, hydrogen production by the electrolysis of water is considered the cleanest way to produce hydrogen, and the purity of the hydrogen produced is relatively higher than that of other methods [3,5]. At present, the highly expensive noble metal-based materials such as platinum (Pt) and RuO_2_ or IrO_2_ are considered ideal catalysts for the HER and OER [6], but their limited reserves, high cost, and poor durability limit the large-scale application of highly expensive noble metal-based materials [7]. Therefore, it is urgent to develop a highly efficient, low-priced, and stable non-noble metal electrocatalyst for the large-scale production of hydrogen.

In recent decades, transition metal-based compounds such as carbides [8,9,10,11], phosphides [12,13,14], sulfides [15,16,17], oxides [18,19,20], borides [21,22,23], and nitrides [24,25,26] have been reported to be used in the HER electrocatalysts and shown good catalytic performance. Among them, molybdenum carbide (Mo_x_C) has attracted extensive attention because of its different Mo valence, adjustable phase composition, high abundance, high chemical stability, and d-band electronic structure, which is similar to Pt-based catalysts [27,28,29,30]. However, single MoC particles are prone to agglomerate at high temperatures, and the hydrogen adsorption capacity of pure MoC is so strong that the conductivity is poor [31], which affects its performance for the HER. Therefore, we can further modify MoC-based catalysts by increasing active sites and introducing carbon supports.

Firstly, it has to be mentioned that, in addition to transition metal carbides, transition metal phosphides are often used as catalysts for the HER because P has lone pair electrons on 3p orbitals and vacant 3d orbitals, which can jointly induce surface charge density. Among many transition metal phosphides, nickel phosphide has attracted much attention because of its special electronic structure and excellent chemical stability [32,33,34,35,36]. According to previous research, the addition of Ni_2_P can increase the electrochemical active surface area (ECSA) and the inherent HER activity of the catalyst [33,37]. The synergistic effect between different phases can achieve goals that cannot be achieved by a single component. Therefore, the performance of the catalyst can be further improved.

Furthermore, to avoid the aggregation of catalyst nanoparticles during high-temperature carbonization, carbon supports such as carbon nanotubes (CNTs) [37], carbon fibers (CFs) [38,39], graphene [40], and carbon black [41] can be introduced. At the same time, these carbon supports can provide a larger specific surface area for the catalyst and expose more catalytic active sites. Furthermore, the precursor of the catalyst encapsulated in CFs prepared by electrospinning technology has limited mobility on the surface of CFs, which can effectively inhibit the aggregation of catalyst nanoparticles [39,42,43]. Among different kinds of carbon materials, coal stands out as an important fossil energy [44]. Transforming it into coal-based carbon fiber (C-CFs) will improve the utilization rate and the market prospect of coal resources [45]. Additionally, the introduction of coal will cause more defects in CFs [46]. For example, researchers have developed a method of applying coal to catalysts for fuel cells and battery materials [46,47]. Moreover, the self-supporting C-CF electrode not only simplifies the electrode preparation process but also avoids the coating falling off of the electrode and the burying of active sites by the adhesive. At the same time, CFs containing metal carbides can be used as conductive bases to grow other active substances so as to further improve the performance of the HER of the materials.

Herein, the Mo source and oxidized coal were uniformly dispersed in the support of the C-CFs by electrospinning, the Ni source was introduced by the impregnation method, and the self-supporting catalysts of Ni_2_P-MoC/C-CFs were obtained by low-temperature phosphorization and high-temperature carbonization. The catalytic performance for the HER of as-prepared Ni_2_P-MoC/C-CFs was improved greatly, and a low overpotential of 112 mV at 10 mA cm^−2^ was exhibited stably. The above results show that it is a promising catalyst for the HER.

## 2. Results and Discussion

### 2.1. Physical Characterization

**Scheme 1:**
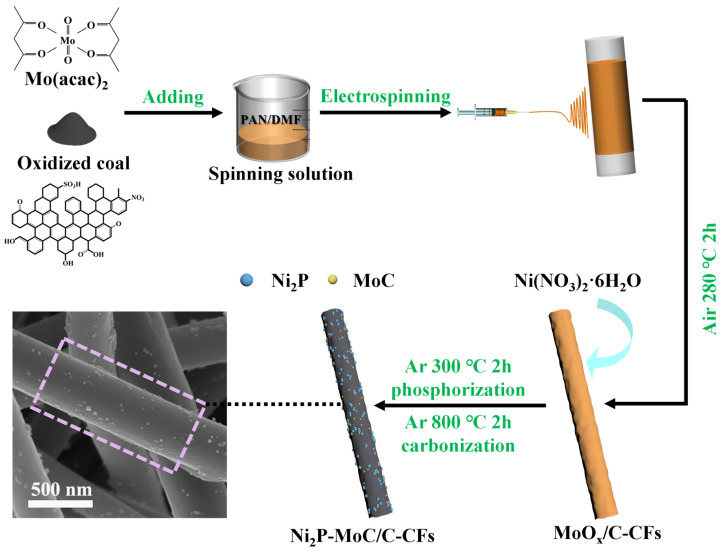
The synthesis procedure of the Ni_2_P-MoC/C-CF electrocatalyst.

**Figure 1:**
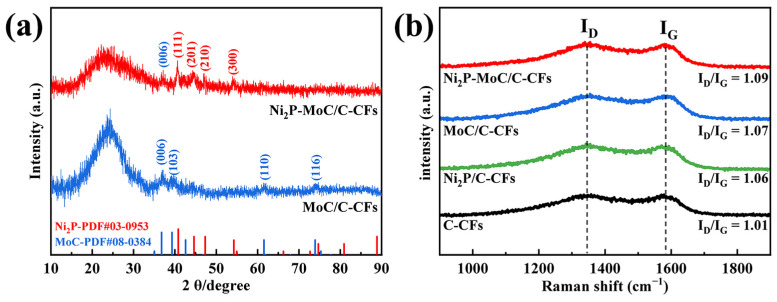
(**a**) X-ray diffraction of Ni_2_P-MoC/C-CFs and MoC/C-CFs. (**b**) Raman spectra of Ni_2_P-MoC/C-CFs, MoC/C-CFs, Ni_2_P/C-CFs, and C-CFs.

The synthetic route for Ni_2_P-MoC/C-CFs is presented in Scheme 1. In this work, using oxidized coal and PAN as carbon sources, molybdenyl acetylacetonate as the Mo source was added to the spinning solution, and a precursor containing MoO_x_ was obtained through electrospinning and pre-oxidation. Then nickel nitrate hexahydrate as the Ni source was introduced into the precursor through impregnation. Using NaPO_2_H_2_·H_2_O as the P source, Ni_2_P was obtained by phosphorization at 300 °C for 2 h, followed by carbonization at 800 °C for 2 h to obtain MoC and C-CFs, ultimately resulting in Ni_2_P-MoC/C-CF composite materials. Figure 1a shows the XRD patterns of the obtained Ni_2_P-MoC/C-CF sample and MoC/C-CF comparison sample. A broad peak located at about 2θ = 23.8° in all catalysts is associated with C-CF support materials, indicating that the carbon material is mostly amorphous carbon. The diffraction peaks at 36.8° and 39.3° can be indexed to the (006) and (103) diffraction planes of MoC (PDF # 08-0384). The diffraction peaks at 40.8°, 44.6°, 47.3°, and 54.2° can be attributed to the (111), (201), (210), and (300) diffraction planes of Ni_2_P (PDF # 03-0953). The XRD results indicate that Ni_2_P-MoC/C-CFs have been successfully synthesized.

Raman spectroscopy was adopted to study the surface structure of different CF materials. Figure 1b compares the Raman test results of the Ni_2_P-MoC/C-CF sample, as well as the MoC/C-CF, Ni_2_P/C-CF, and C-CF comparison samples. The two strong peaks between 1330 and 1580 cm^−1^ correspond to defect characteristics (D band) and graphitization degrees (G band), respectively. In general, the D band reflects defects in the crystal structure, and the G band represents graphitelike carbon [48]. The larger the intensity ratios from the D peak to the G peak (I_D_/I_G_) are, the more defects and exposed edges there are in the carbon material, which is conducive to electron transfer in the electrochemical reaction process [49]. The I_D_/I_G_ ratios of Ni_2_P-MoC/C-CFs, MoC/C-CFs, Ni_2_P/C-CFs, and C-CFs are calculated to be 1.09, 1.07, 1.06, and 1.01, respectively. The Ni_2_P-MoC/C-CFs have the maximum I_D_/I_G_ ratio, which shows that the introduction of Ni_2_P and MoC increases the number of structural defects in the carbon lattice and reduces the order degree of C-CFs. The results suggest that transition metal elements play a prominent role in facilitating carbon graphitization and higher electrical conductivity in carbon materials, leading to better efficiency. Furthermore, Figure S1 shows that the I_D_/I_G_ ratio of Ni_2_P-MoC/C-CFs (1.09) is higher than that of Ni_2_P-MoC-CFs (1.05). This may be attributed to the fact that the addition of oxidized coal reduces the ordered arrangement of PAN chains in CFs, which leads to more structural defects.

**Figure 2:**
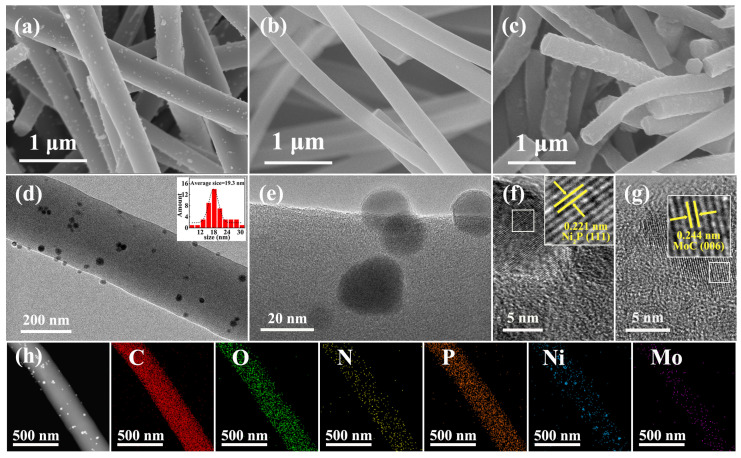
SEM image of (**a**) Ni_2_P-MoC/C-CFs, (**b**) MoC/C-CFs, and (**c**) Ni_2_P/C-CFs; (**d**–**g**) TEM and HRTEM images of Ni_2_P-MoC/C-CFs and (**h**) the corresponding element mapping images of C, O, N, P, Ni, and Mo.

As shown in Figure 2, the microstructures and morphologies of the samples were characterized by scanning electron microscopy (SEM) and transmission electron microscopy (TEM). Figure 2a–c shows SEM images of Ni_2_P-MoC/C-CFs, MoC/C-CFs, and Ni_2_P/C-CFs, respectively. It can be seen from Figure 2a and Figure S2 that the nanoparticles are uniformly grown on C-CFs, and the average diameter of the nanoparticles is about ~19.3 nm (inset in Figure 2d). In Figure 2b and Figure S3, the rough surfaces of MoC/C-CFs endow the self-supporting electrode with a large specific surface area and provide more active sites. In comparison with Ni_2_P-MoC/C-CFs, the nanoparticle size of Ni_2_P/C-CFs is obviously increased, and the growth was uneven and agglomerated (Figure 2c and Figure S4). This shows that the addition of the Mo source has a certain dispersion and anchoring effect on Ni_2_P nanoparticles grown on the C-CFs surface. Figure 2f,g shows that the lattice spacing of the nanoparticles on the C-CFs at 0.221 and 0.244 nm is consistent with the (111) crystal planes of Ni_2_P and (006) crystal planes of MoC, respectively, which further shows that Ni_2_P and MoC nanoparticles were successfully grown on C-CFs. In addition, the EDS-mapping image (Figure 2h) shows the uniform distribution of C, O, N, P, Ni, and Mo elements in Ni_2_P-MoC/C-CFs.

**Figure 3:**
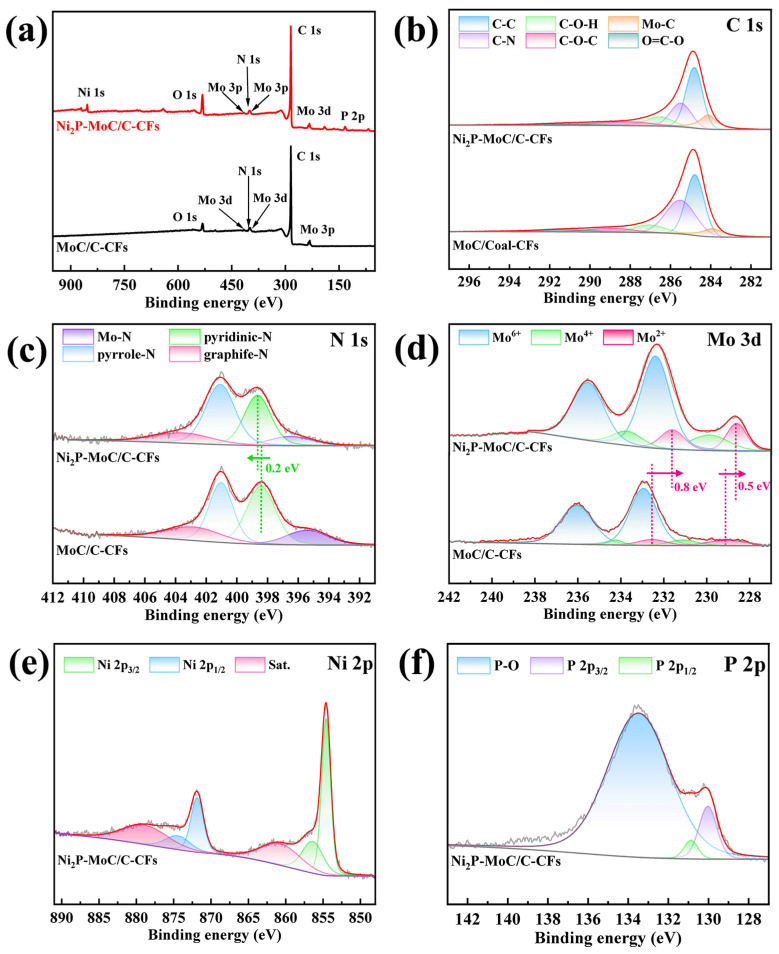
(**a**–**d**) XPS Survey spectrum and high-resolution XPS C 1s, N 1s, and Mo 3d spectra of Ni_2_P-MoC/C-CFs and MoC/C-CFs; (**e**,**f**) high-resolution XPS Ni 2p and P 2p spectra of Ni_2_P-MoC/C-CFs.

X-ray photoelectron spectroscopy (XPS) analysis was carried out to understand the surface electronic conditions and chemical states of the samples before and after the introduction of Ni_2_P. In Figure 3a, the full spectra of as-prepared catalysts exhibit the chemical signals of Mo, C, N, O, Ni, and P elements in the Ni_2_P-MoC/C-CF sample and Mo, C, N, and O elements in the MoC/C-CF comparison sample. In the C 1s XPS spectrum (Figure 3b), six peaks at 284.0, 284.8, 285.6, 286.6, 288.2, and 291.0 eV are assigned to Mo-C, C-C, C-N, C-O-H, C-O-C, and O=C-O, respectively, revealing that impregnation treatment shows no distinct influence on the chemical state of C species. The appearance of the C-N peak and Mo-C peak indicates the successful doping of N in the C-CFs and the existence of MoC, respectively.

The high-resolution N 1s spectrum (Figure 3c) of Ni_2_P-MoC/C-CFs and MoC/C-CFs can be deconvoluted into four peaks, corresponding to Mo-N bond (~396.5 eV), pyridine-N (~398.65 eV), pyrrole-N (~401.1 eV), and graphite-N (~403.7 eV), respectively. The molecular model structures of these three types of N are shown in Figure S5. The doping of N atoms in C-CFs not only affects the electronic structure of MoC but also improves the chemical stability. The peaks of Mo 3d in Ni_2_P-MoC/C-CFs and MoC/C-CFs could be deconvoluted into three doublets, corresponding to the three valence states (+2, +4, and +6) of Mo on the surface. The Mo^2+^ peaks at 228.6 and 231.6 eV, respectively, are attributed to the Mo-C bond and are generally considered to be the electrocatalytic active centers [28]. Herein, the existence of the Mo^4+^ and Mo^6+^ species can be attributed to the surface oxides formed during the pre-oxidation process [50,51]. Compared with MoC/C-CFs, these two peaks about Mo^2+^ were negatively shifted by approximately 0.5 and 0.8 eV, respectively (Figure 3d), suggesting enriched electrons around the Mo species [50]. In addition, Mo^4+^ and Mo^6+^ showed a similar phenomenon. Generally, an increase in the valence electron charge results and a reduction in binding energy were observed [52]. Therefore, the negative binding energy shifts of the Mo 3d can be attributed to the transfer of valence electrons from Ni to MoC across their interfaces. This efficient electron transfer contributes to a synergistic effect on Ni_2_P-MoC/C-CFs, which diminishes the hydrogen binding energy and accordingly contributes to the desorption of hydrogen adsorbate (H_ads_) and H_2_ generation [52,53]. The characteristic peaks of Ni 2p_3/2_, Ni 2p_1/2_, and satellite peaks can be observed in the high-resolution Ni 2p spectrum (Figure 3f). The Ni 2p_3/2_ and Ni 2p_1/2_ peaks can be decomposed into two parts: the peaks at 854.6 and 871.88 eV are attributed to metal Ni, and the peaks at 856.46 and 874.6 eV are attributed to Ni^2+^. The peaks at 861.14 and 879.32 eV are satellite peaks. In addition, the peak positions of P 2p_3/2_, P 2p_1/2_, and oxidized P species displayed in the P 2p (Figure 3e) spectrum are 130.05, 130.9, and 133.5 eV, respectively. The P-O peak at 133.5 eV is due to the inevitable surface oxidation of the sample exposed to air, which can be attributed to the high oxidation state of P species (PO_x_) [32,54,55].

**Figure 4:**
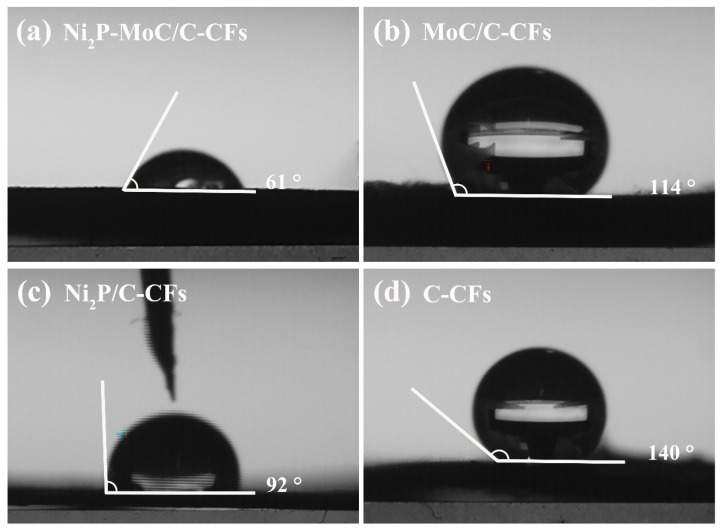
Water droplet contact angles on (**a**) Ni_2_P-MoC/C-CFs, (**b**) MoC/C-CFs, (**c**) Ni_2_P/C-CFs, and (**d**) C-CFs.

Previous works have demonstrated that the super-hydrophilic surface of the electrocatalyst is beneficial for increasing the contact area of the catalyst with the electrolyte and H_2_ bubbles released from the electrode. The compatibility with water and the charge transfer has thus been remarkably enhanced [56]. Figure 4 shows that the water contact angles of Ni_2_P-MoC/C-CFs, MoC/C-CFs, Ni_2_P/C-CFs, and C-CFs at the same time interval are 61°, 114°, 92°, and 140°, respectively. In comparison with Ni_2_P-MoC/C-CFs and Ni_2_P/C-CFs, the hydrophilic effect of MoC/C-CFs and C-CFs is decreased significantly, which is attributed to the introduction of the Ni source by the impregnation treatment. After the impregnation treatment, the content of the pyridine-N species significantly increases, and the more pyridine-N there is on the surface, the more conducive it is to improve the surface wettability of the material [56,57]. This is consistent with the XPS results.

### 2.2. Electrochemical Properties

A typical three-electrode system was used to test the performance of the HER of a series of catalysts in an N_2_-saturated acid electrolyte (0.5 M H_2_SO_4_ with a pH of 0.3). The effects of the Ni source impregnation amount and the introduction of oxidized coal improve the performance of the HER of the materials that were studied.

**Figure 5:**
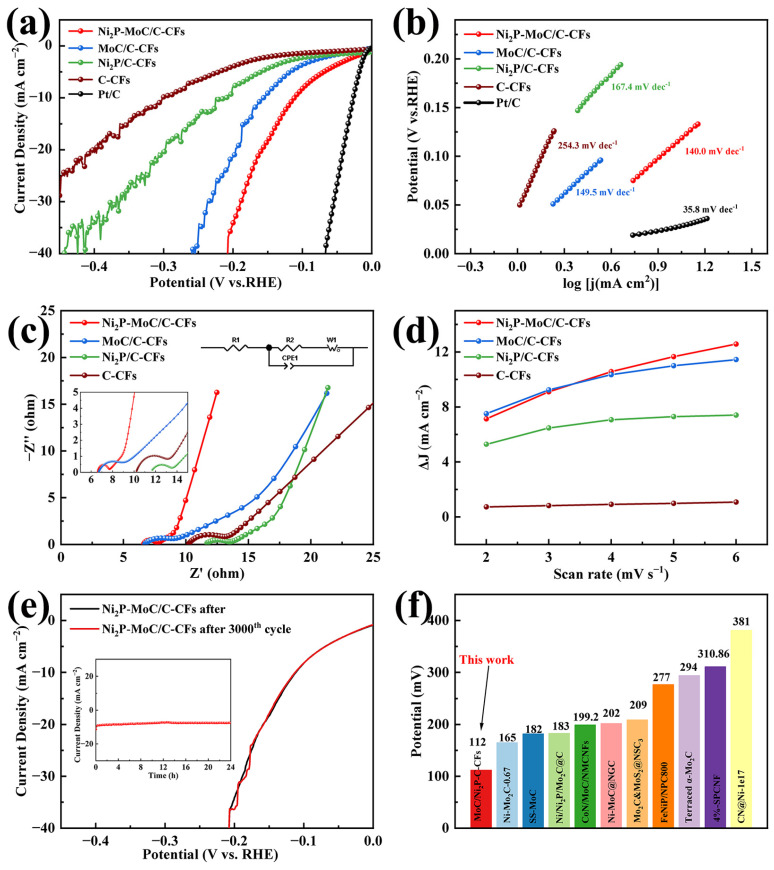
The electrocatalytic measures of Pt/C, Ni_2_P-MoC/C-CF, MoC/C-CF, Ni_2_P/C-CF, and C-CF (**a**) LSV polarization curves; (**b**) Tafel diagram; the electrocatalytic measures of Ni_2_P-MoC/C-CF, MoC/C-CF, Ni_2_P/C-CF, and C-CF (**c**) Nyquist diagrams; (**d**) C_dl_ measurement; (**e**) the LSV polarization curve of Ni_2_P-MoC/C-CFs before and after 3000 cycles and 24 h long-term durability testing (inset); and (**f**) overpotential histograms of various reported HER catalysts.

As shown in Figure S6, the crossing points of Ni_2_P-MoC/C-CFs(1:1), Ni_2_P-MoC/C-CFs(1:2), and Ni_2_P-MoC/C-CFs(1:3) at 10 mA cm^−2^ current density are 165, 112, and 142 mV, respectively. It shows that the best molar ratio between the Mo and Ni source is 1:2. Correspondingly, Figure S7 compares the properties of the samples with (Ni_2_P-MoC/C-CFs) or without oxidized coal (Ni_2_P-MoC-CFs) and proves that the introduction of oxidized coal makes the material contain more oxygen-containing functional groups, which makes the material have better performance for the HER. Figure 5a compares the LSV curves of 20% Pt/C, Ni_2_P-MoC/C-CFs, MoC/C-CFs, Ni_2_P/C-CFs, and C-CFs at a scanning rate of 2 mV s^−1^. The overpotential of Ni_2_P-MoC/C-CFs at a current density of 10 mA cm^−2^ is only 112 mV, which is superior to the 155 mV of MoC/C-CFs, 210 mV of Ni_2_P/C-CFs, and 301 mV of C-CFs and close to 27 mV of commercial Pt/C. After the combination of MoC with Ni_2_P, the overpotentials are decreased significantly, indicating that the synergistic effect between MoC and Ni_2_P can improve the activity for the HER of the Ni_2_P-MoC/C-CF self-supporting electrode. Then, the Tafel slope is used to further analyze the process. Accordingly, the Tafel plots (Figure 5b) derived from the polarization curves imply that the Ni_2_P-MoC/C-CF electrode has a relatively small Tafel slope value of 140.0 mV dec^−1^, which is only greater than Pt/C among all electrolytic samples (149.6 mV dec^−1^ for MoC/C-CFs, 167.4 mV dec^−1^ for Ni_2_P/C-CFs, and 254.3 mV dec^−1^ for C-CFs). This indicates that Ni_2_P-MoC/C-CFs have the most favorable kinetics for the HER. Generally, the semicircle in the low-frequency range of the Nyquist plots is related to the charge-transfer resistance R_ct_, while the solution resistance R_s_ is implicit in the high-frequency range [58]. As shown in Figure 5c, Ni_2_P-MoC/C-CFs revealed the R_ct_ of 6.26 Ω, smaller than the R_ct_ of MoC/C-CFs (6.91 Ω), Ni_2_P/C-CFs (9.95 Ω), and C-CFs (13.58 Ω), which indicated a significantly faster reaction rate for the HER. In addition, the electrochemical specific surface area (ECSA) of the electrocatalyst was measured to understand the source of the HER activity enhancement, which was proportional to the double-layer capacitance (C_dl_). In this work, the C_dl_ was determined by cyclic voltammetry (CV) at diverse scan rates (2–6 mV s^−1^) in the non-faradaic potential range (Figure 5d). Figures S8–S10 is a typical cyclic voltammogram of different samples at different scanning rates. The C_dl_ value of Ni_2_P-MoC/C-CFs is estimated to be 1342.2 mF cm^−2^ by fitting the capacitive currents with the scan rates, greatly larger than that of MoC/C-CFs (962.2 mF cm^−2^), Ni_2_P/C-CFs (506.7 mF cm^−2^), and C-CFs (84.3 mF cm^−2^), suggesting that the higher catalytic activity for the HER of Ni_2_P-MoC/C-CFs is relevant to its larger ECSA. The HER stability of Ni_2_P-MoC/C-CF in 0.5 M H_2_SO_4_ was tested. In Figure 5e, after 3000 cycles of CV test, the LSV curve of Ni_2_P-MoC/C-CFs is almost the same as the initial curve. Furthermore, the long-term stability of the catalyst investigated by a potentiostatic method showed that the current density remains almost unchanged within 24 h (Figure 5e inset). These results indicated that Ni_2_P-MoC/C-CFs had good stability. It is worth noting that, as shown in Figure 5f and Table S1, the catalytic activity of the Ni_2_P-MoC/C-CF electrode is equivalent to or even better than that of the non-noble catalysts reported recently. In addition, the XRD, SEM, and XPS of the Ni_2_P-MoC/C-CF catalyst were also tested after the long-term stability test. The XRD results in Figure S11 show that after the long-term stability test, the XRD peak of Ni_2_P in the Ni_2_P-MoC/C-CF catalyst has been reduced to a certain extent, and the crystallinity of Ni_2_P has decreased, but the corresponding peak of Ni_2_P can still be observed. After long-term stability testing, the MoC species were least affected and the XRD peaks remained almost unchanged. The SEM images show that after 24 h i-t testing, the nanoparticles on the surface of Ni_2_P-MoC/C-CFs tend to expand, which may be attributed to the aggregation phenomenon of the catalyst after the HER. In addition, comparing XPS before and after 24 h i-t testing (Figure S13), the Mo^6+^ peaks at 232.4 and 235.5 eV decreased, while the peaks of Ni^2+^ at 856.46 and 874.6 eV increased, indicating that some of the zero-valent nickel transformed into high-valent nickel and that Ni electrons transferred to Mo. In conclusion, the coupling of Ni_2_P and MoC makes the Ni_2_P-MoC/C-CF self-supporting electrode obtain excellent activity and kinetics for the HER.

## 3. Materials and Methods

### 3.1. Materials

The following materials were used: raw coal (Heishan, Xinjiang, China), polyacrylonitrile (PAN, analytical purity (AR), Beijing Bailingwei Technology Co., Ltd., China. MW = 150,000), *N*,*N*-dimethylformamide (DMF, AR, Tianjin Xinbote Chemical Co., Ltd., Tianjin, China), concentrated nitric acid (AR, Tianjin Fengchuan Chemical Reagent Technology Co., Ltd., Tianjin, China), Mo(acac)_2_ (98%; Adamas-Beta) Ni(NO_3_)_2_ 6H_2_O (Shanghai Aladdin Reagent Co., Ltd., Shanghai, China), and NaPO_2_H_2_·H_2_O (99%, Shanghai Aladdin Reagent Co., Ltd., China).

### 3.2. Acidification Treatment of Coal

Firstly, we sieved the raw coal with a 200-mesh sieve, took 10 g of the screened coal powder, and placed it in a 5 L beaker. We mixed 180 mL of concentrated sulfuric acid (98%) and 60 mL of concentrated nitric acid (68%), cooled to room temperature, and slowly poured them into a beaker bathed in ice water. After the mixture reached room temperature, we added DI water and let it settle. We rinsed the lower sediment of the turbid solution twice with 5 L of DI water. The oxidized coal obtained after filtration was dried in an air-blast drying furnace at 80 °C for 12 h.

### 3.3. Synthesis of Precursor

We added 0.5 mmol of Mo(acac)_2_ to 10 mL DMF, stirred evenly, and then added 0.8 g of PAN and 0.8 g of oxidized coal and stirred overnight. We poured the spinning solution into a 10 mL needle tube and connected the No. 20 stainless steel needle. The voltage and distance between the needle tip and the collector were 18 kV and 15 cm, respectively, and the propulsion speed was 0.8 mL h^−1^. We collected the initially spun fibers on a drum wrapped in aluminum foil. The obtained initial spun fibers were heated in a muffle furnace to 280 °C and maintained for 2 h (heating rate: 1 °C min^−1^) to obtain the precursors.

### 3.4. Synthesis of Independent Self-Supporting Catalyst

We set the dimensions to 2 × 4 cm, and the precursor was immersed in 20 mL DI water containing 1, 2, and 3 mmol Ni(NO_3_)_2_·6H_2_O for 12 h. After removal, the precursor was rinsed with DI water and ethanol three times. Vacuum drying was performed at 60 °C for 10 h. The precursor was placed downstream of the porcelain boat, and NaPO_2_H_2_·H_2_O was placed upstream of the porcelain boat as the phosphorus source. We carefully removed the air by pumping three times (using Ar). Annealing was performed at 300 °C for 2 h (heating rate: 1 °C min^−1^) and carbonizing at 800 °C for 2 h (heating rate: 5 °C min^−1^) with Ar. The synthesized products were named Ni_2_P-MoC/C-CFs (1:1), (1:2), and (1:3), according to the content of the impregnated Ni source.

### 3.5. Synthesis Method of Comparison Samples

The precursor synthesized in Section 3.3 was carbonized under an Ar atmosphere at 800 °C for 2 h to obtain the MoC/C-CF comparison sample.

We added 0.8 g PAN and 0.8 g oxidized coal to 10 mL DMF and stirred evenly. We poured the spinning solution into a 10 mL syringe and connected the No. 20 stainless steel needle. The voltage and distance between the needle tip and the collector were 18 kV and 15 cm, respectively, and the electrospinning was carried out at a speed of 0.8 mL h^−1^. The obtained precursor was pre-oxidized in an atmosphere of 280 °C. Subsequently, carbonization was carried out in an Ar atmosphere at 800 °C for 2 h to obtain C-CF comparison samples.

With the dimensions of 2 × 4 cm, C-CFs were immersed in 20 mL DI water containing 1 mmol Ni(NO_3_)_2_·6H_2_O for 12 h. After removal, the sample were rinsed with DI water and ethanol three times. Vacuum drying was performed at 60 °C for 10 h. The precursor was placed downstream of the porcelain boat, and NaH_2_PO_2_ was placed upstream of the porcelain boat as the phosphorus source. Carefully, we removed the air by pumping three times (using argon). Then annealing was performed at 300 °C for 2 h (heating rate: 1 °C min^−1^) and carbonization was performed at 800 °C for 2 h (heating rate: 5 °C min^−1^) to obtain the Ni_2_P/C-CF comparison sample.

### 3.6. Characterizations

We determined the phase and composition structure of the sample through X-ray diffraction (XRD, German, BRUKER D8 Advanced); Raman spectra were taken on a Raman spectrometer (Raman, France, HORIBA Lab RAM HR Evolution). The morphology and structure of the samples were characterized by field emission scanning electron microscopy (SEM, Japan, Hitachi, SU8010) and field emission high-power transmission electron microscopy (HRTEM, Japan, JEM-2100F). The surface elemental composition and valence states of the samples were studied by X-ray photoelectron spectroscopy (XPS, Waltham, MA, USA, Escalable 250Xi from Thermo Fisher Scientific). The hydrophilicity and hydrophobicity of the samples were determined by an optical contact angle tester (Shanghai, China, Powereach, JJ200B2).

### 3.7. Electrochemical Measurements

Electrochemical measurements were performed on a CHI 760E electrochemical workstation with a three-electrode system. The 0.5 × 1 cm self-supporting catalyst was used as the working electrode, carbon rod as the counter electrode, and Ag/AgCl as the reference electrode. The electrolyte is a 0.5M H_2_SO_4_ solution with a pH of 0.3. Before each electrochemical measurement, we ventilated with high-purity N_2_ for 30 min to remove dissolved O_2_ from the electrolyte. During the test, we stopped stirring and continued to apply N_2_. We performed cyclic voltammetry (CV) curve testing at a voltage window of −0.2–−0.1 V and a scanning rate of 100 mV s^−1^. We recorded the linear sweep voltammetry (LSV) curve at a scanning rate of 2 mV s^−1^ and a potential range of −0.80–−0.10 V. We converted all potentials to reversible hydrogen electrodes (RHE) using the equation E_(RHE)_ = E_(Ag/AgCl)_ + 0.059 pH + 0.2046 and corrected with 90% iR compensation. The Tafel plot was calculated from the corresponding LSV curve. The double-layer capacitance (C_dl_) was estimated from the CV curves in the unlawfully charged potential region (0.022–0.122V) at different scanning rates (2–6 mV s^−1^).

## 4. Conclusions

In summary, the Mo source and oxidized coal were uniformly dispersed in the carbon support by electrospinning technology. The Ni source was introduced by the impregnation method. Highly efficient non-noble-metal self-supporting catalyst Ni_2_P-MoC/C-CFs were obtained after low-temperature phosphorization and high-temperature carbonization. It is worth noting that due to the uniform distribution of the Mo species in C-CFs, Ni_2_P nanoparticles grow evenly on the surface of C-CFs. As a result, the catalytic performance of the HER was improved greatly, and a low overpotential of 112 mV at 10 mA cm^−2^ was exhibited stably by the Ni_2_P-MoC/C-CF material in an acidic electrolyte. In addition, the results demonstrate that the excellent electrochemical performances of the self-supporting electrodes are attributed to the electron interaction between Ni_2_P and MoC as well as the unique electrode structure without the usage of any binder. Therefore, this research has made a breakthrough in the development of efficient and low-cost self-supporting catalysts for the HER, brightening the prospects for its application.

## Data Availability

Additional data are made available in the Supplementary Materials of this manuscript.

## Supplementary Materials

The following supporting information can be downloaded at: https://www.mdpi.com/article/10.3390/molecules29010116/s1, Figure S1. Raman spectra of the prepared Ni_2_P-MoC/C-CFs and Ni_2_P-MoC-CFs. Figure S2. SEM of Ni_2_P-MoC/C-CFs at different magnifications. Figure S3. SEM of MoC/C-CFs at different magnifications. Figure S4. SEM of Ni_2_P/C-CFs at different magnifications. Figure S5. schematic diagram of different N types. Figure S6. The electrocatalytic measures of Ni_2_P-MoC/C-CF(1:1), Ni_2_P-MoC/C-CF(1:2), and Ni_2_P-MoC/C-CF(1:3) (a) LSV polarization curves; (b) Tafel diagram, (c) Nyquist diagram, and (d) Cdl measurement. Figure S7. The electrocatalytic measures of Ni_2_P-MoC/C-CF and Ni_2_P-MoC-CF (a) LSV polarization curves; (b) Tafel diagram; (c) Nyquist diagram and (d) Cdl measurement. Figure S8. Typical cyclic voltammograms at different scan rates for (a) Ni_2_P-MoC/C-CFs (1:1); (b) Ni_2_P-MoC/C-CFs (1:2), and (c) Ni_2_P-MoC/C-CFs (1:3) with scan rates ranging from 2 mV s^−1^ to 6 mV s^−1^ in 0.5 M H_2_SO_4_. Figure S9. Typical cyclic voltammograms at different scan rates. (a) Ni_2_P-MoC/C-CFs and (b) Ni_2_P-MoC-CFs with scan rates ranging from 2 mV s^−1^ to 6 mV s^−1^ in 0.5 M H_2_SO_4_. Figure S10. Typical cyclic voltammograms at different scan rates. (a) Ni_2_P-MoC/C-CFs; (b) MoC/C-CFs; (c) Ni_2_P/C-CFs, and (d) C-CFs with scan rates ranging from 2 mVs^−1^ to 6 mV s^−1^ in 0.5 M H_2_SO_4_. Figure S11. XRD characterization of Ni_2_P-MoC/C-CFs catalysts before and after long-term stability testing. Figure S12. SEM image of Ni_2_P-MoC/C-CFs catalysts before and after long-term stability testing. Figure S13. High-resolution XPS (a) Mo 3d and (b) Ni 2p spectra of Ni_2_P-MoC/C-CF catalysts before and after long-term stability testing. Table S1. Comparison of HER activity data for different catalysts. References [42,51,59,60,61,62,63,64,65,66] can be found in the Supplementary Materials.
