# Supplementary material for: Construction of Ni2P-MoC/Coal-Based Carbon Fiber Self-Supporting Catalysts for Enhanced Hydrogen Evolution
Source: Molecules. 2023 Dec 24;29(1):116. doi: 10.3390/molecules29010116 (PMC10779885; doi:10.3390/molecules29010116)
Supplement: Supplementary file 1 [file molecules-29-00116-s001.zip › molecules-2756336-supplementary.pdf]

Supplementary Materials

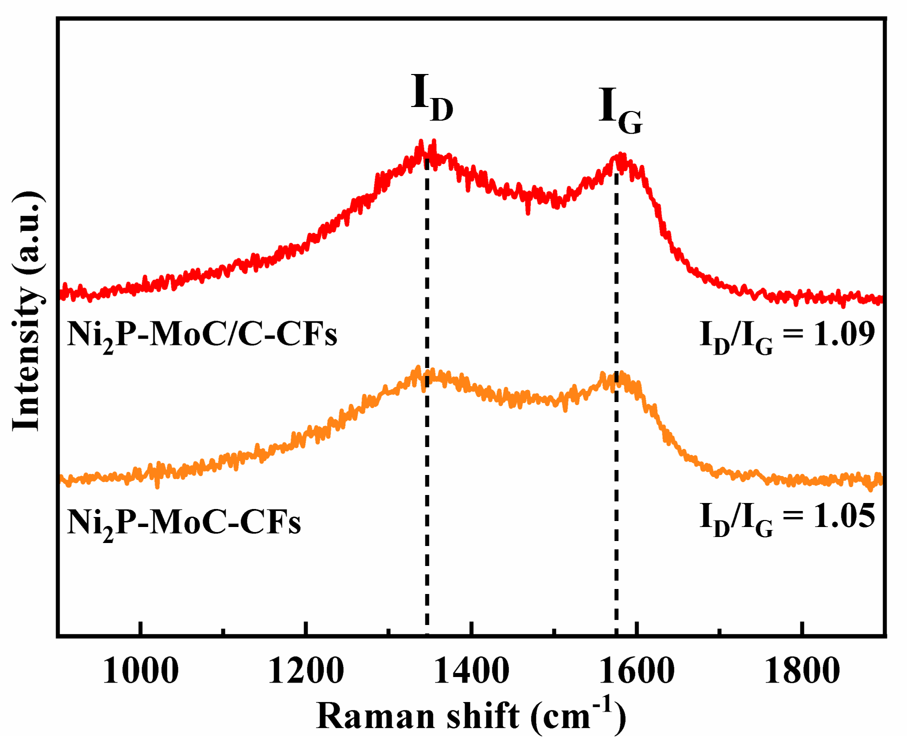

Figure S1. Raman spectra of the prepared Ni_2P-MoC/C-CFs and Ni_2P-MoC-CFs.

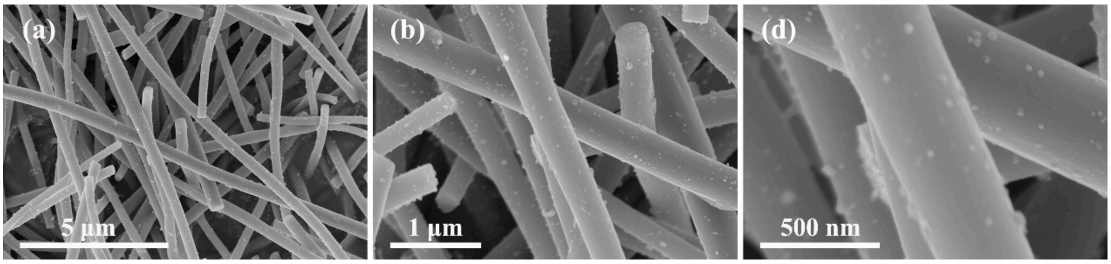

Figure S2. SEM of Ni_2P-MoC/C-CFs at different magnifications.

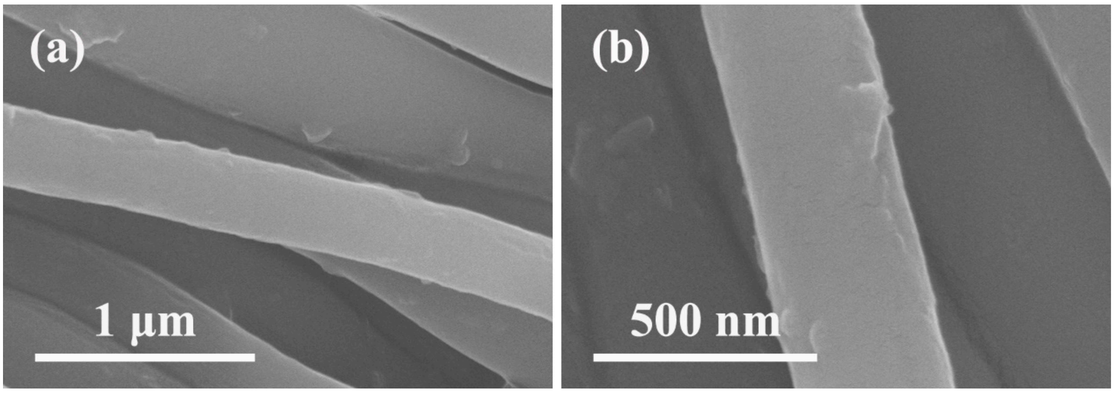

Figure S3. SEM of MoC/C-CFs at different magnifications.

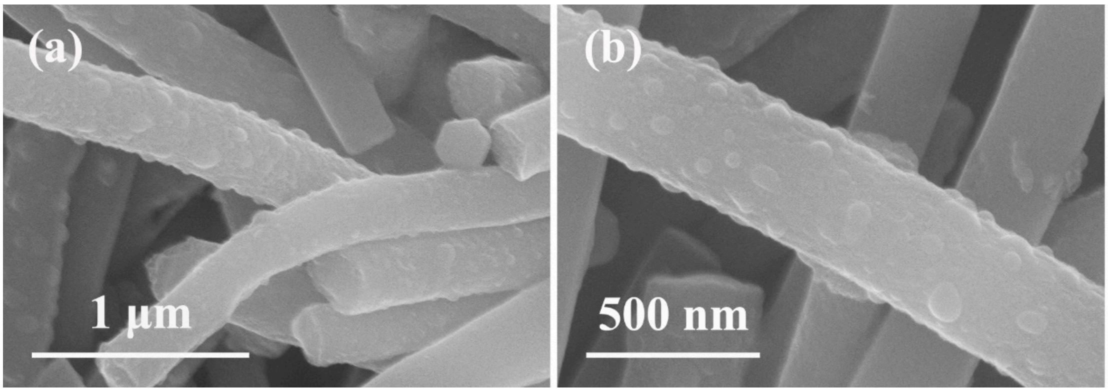

Figure S4. SEM of Ni2P/C-CFs at different magnifications.

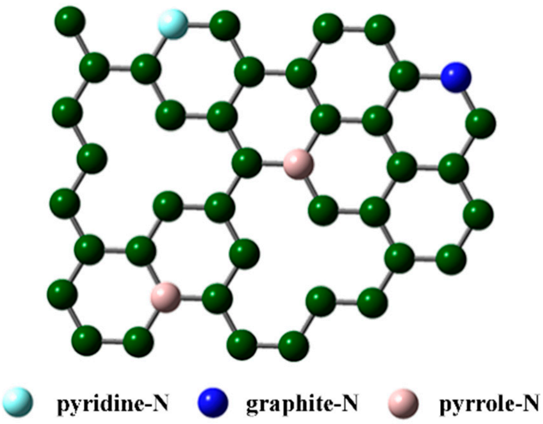

Figure S5. schematic diagram of different N types.

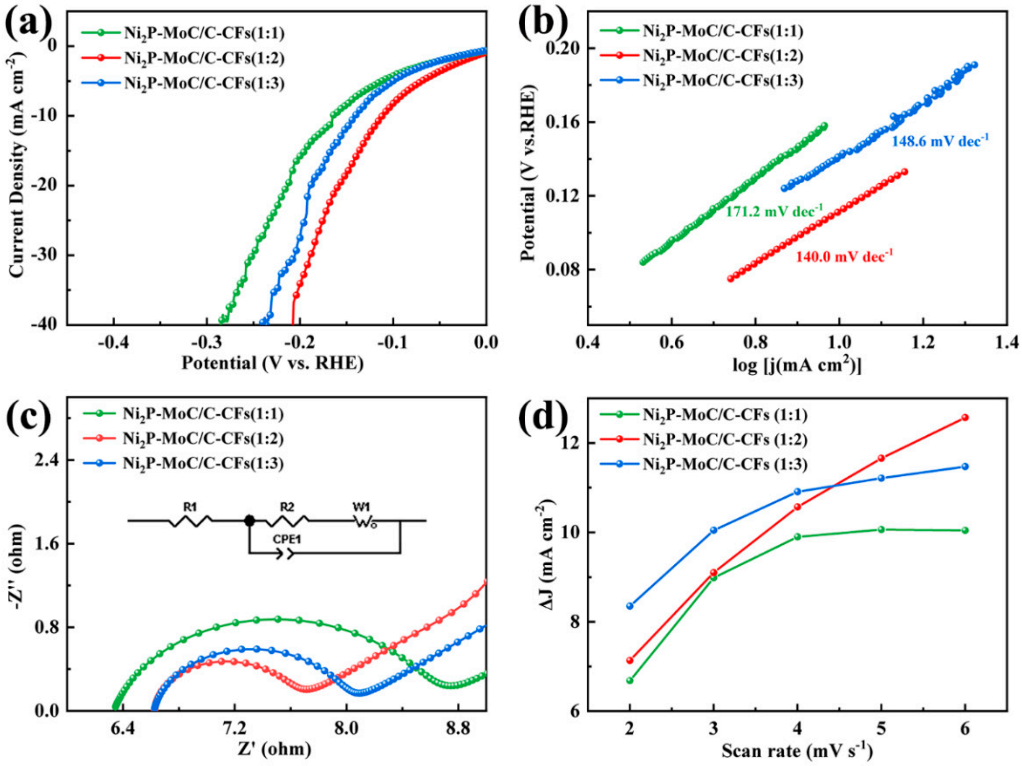

Figure S6. The electrocatalytic measures of Ni2P-MoC/C-CFs(1:1),

Ni_2P-MoC/C-CFs(1:2), and Ni_2P-MoC/C-CFs(1:3) (a) LSV polarization curves; (b) Tafel diagram (c) Nyquist diagram, and (d) C_{dl} measurement.

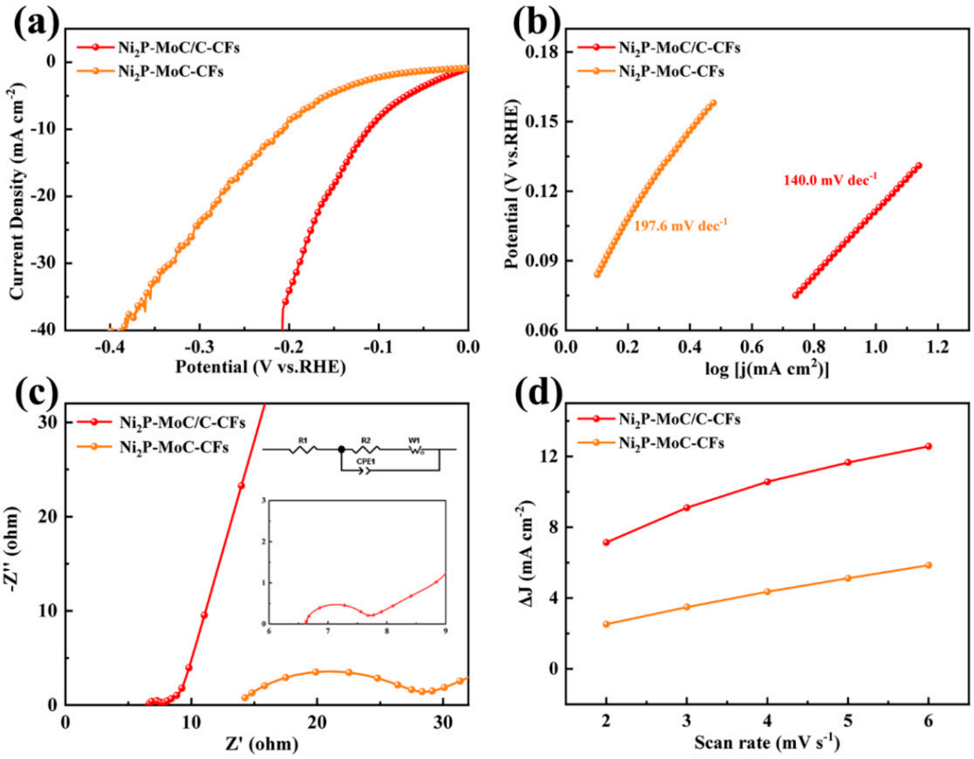

Figure S7. The electrocatalytic measures of Ni_2P-MoC/C-CFs and Ni_2P-MoC-CFs (a) LSV polarization curves; (b) Tafel diagram; (c) Nyquist diagram and (d) C_{dl} measurement.

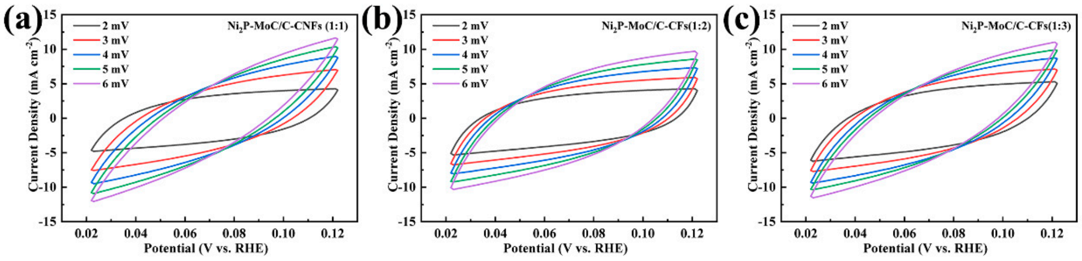

Figure S8. Typical cyclic voltammograms at different scan rates for (a) Ni_2P-MoC/C-CFs (1:1); (b) Ni_2P-MoC/C-CFs (1:2), and (c) Ni_2P-MoC/C-CFs (1:3) with scan rates ranging from 2 mV s^{-1} to 6 mV s^{-1} in 0.5 M H_2SO_4.

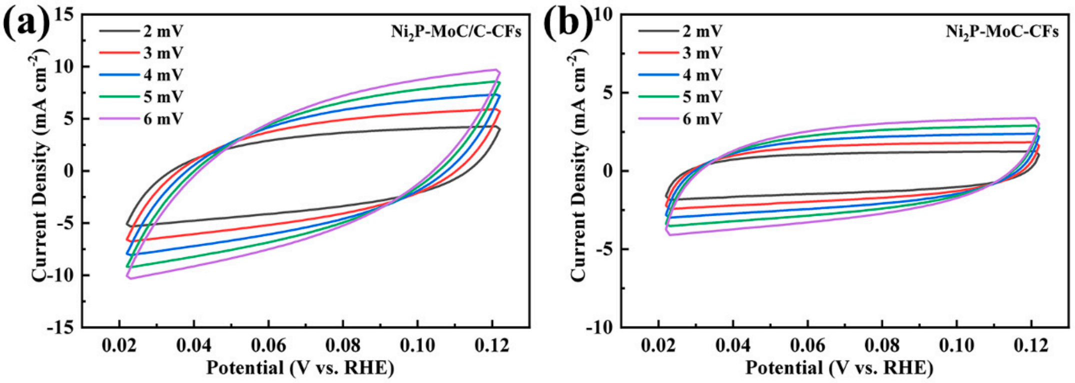

Figure S9. Typical cyclic voltammograms at different scan rates. (a) Ni_2P-MoC/C-CFs and (b) Ni_2P-MoC-CFs with scan rates ranging from 2 mV s^{-1} to 6 mV s^{-1} in 0.5 M H_2SO_4.

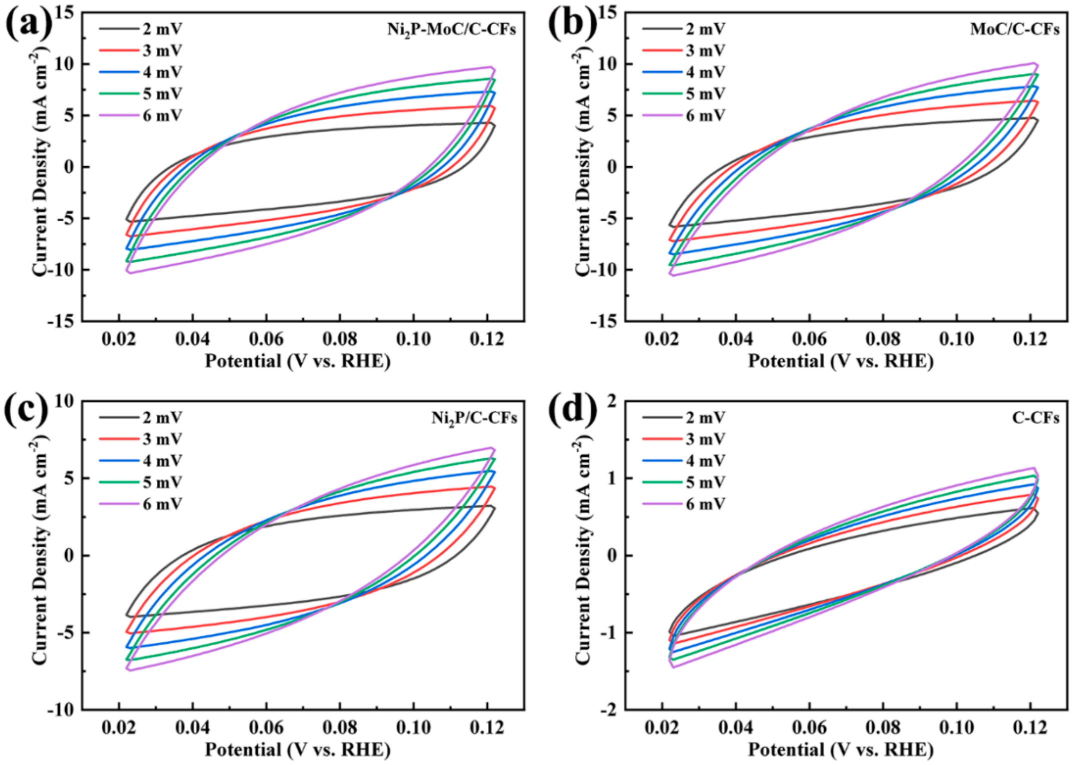

Figure S10. Typical cyclic voltammograms at different scan rates. (a) Ni_2P-MoC/C-CFs; (b) MoC/C-CFs; (c) Ni_2P/C-CFs, and (d) C-CFs with scan rates ranging from 2 mVs^{-1} to 6 mV s^{-1} in 0.5 M H_2SO_4.

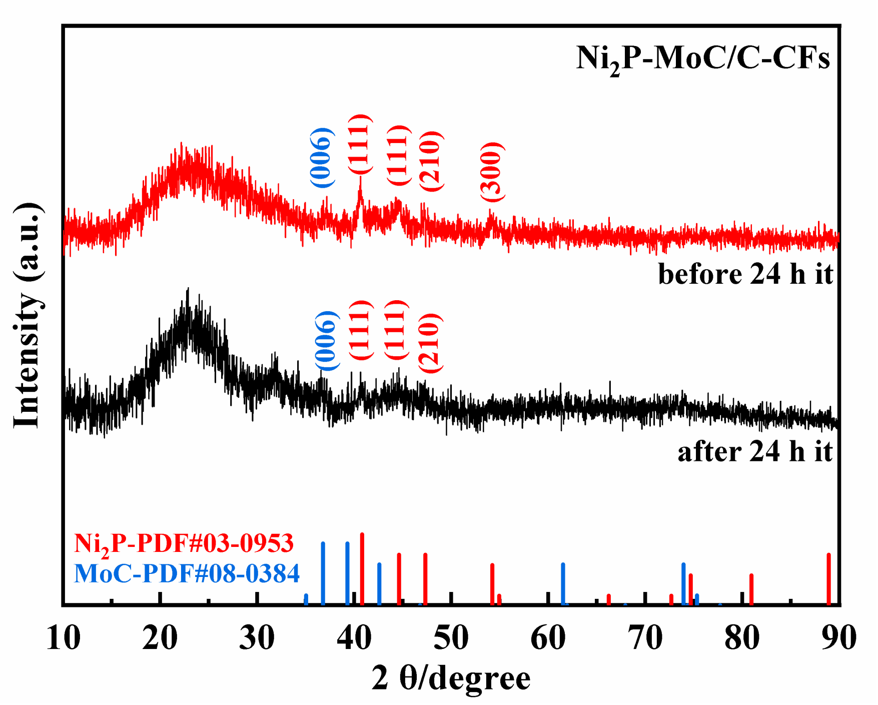

Figure S11. XRD characterization of Ni_2P-MoC/C-CFs catalysts before and after long-term stability test.

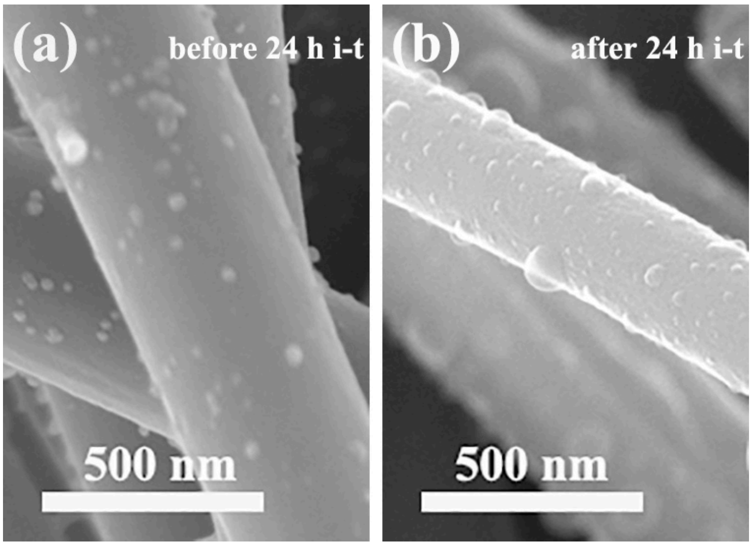

Figure S12. SEM image of Ni_2P-MoC/C-CFs catalysts before and after long-term stability test.

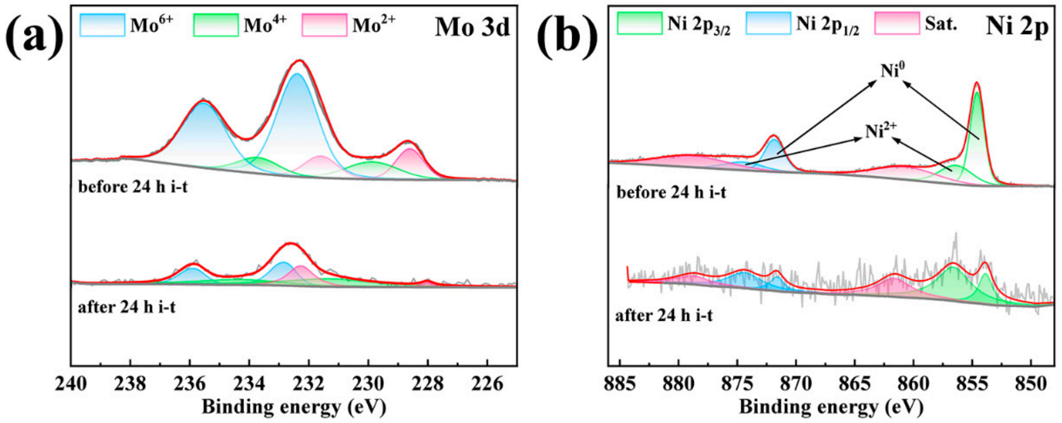

Figure S13. High resolution XPS (a) Mo 3d and (b) Ni 2p spectra of Ni_2P-MoC/C-CFs catalysts before and after long-term stability testing.

Table S1. Comparison of HER activity data for different catalysts.

Material	η_{10} (mV)	Electrolyte	Ref.
MoC/Ni_2P-C-CFs (this work)	112	0.5 M H_2SO_4	This work
Ni-Mo_2C-0.67	165	0.5 M H_2SO_4	[51]
SS-MoC	182	0.5 M H_2SO_4	[59]
Ni/Ni_2P/Mo_2C@C	183	0.5 M H_2SO_4	[60]
CoN/MoC/NMCNFs	199.2	0.5 M H_2SO_4	[42]
Ni-MoC@NGC	202	0.5 M H_2SO_4	[61]
Mo_2C&MoS_2@NSC_3	209	0.5 M H_2SO_4	[62]
FeNiP/NPC800	277	0.5 M H_2SO_4	[63]
Terraced α-Mo_2C	294	0.5 M H_2SO_4	[64]
4%-SPCNF	310.86	0.5 M H_2SO_4	[65]
CN@Ni-1e17	381	0.5 M H_2SO_4	[66]
